# Prevalence and determinants of cigarette smoking among college students: a cross-sectional study in Douala, Cameroon

**DOI:** 10.1186/s13690-015-0100-1

**Published:** 2015-12-21

**Authors:** Bertrand Hugo Mbatchou Ngahane, Huguette Atangana Ekobo, Christopher Kuaban

**Affiliations:** Department of Internal Medicine, Douala General Medicine, PO Box 4856, Douala, Cameroon; Faculty of Medicine and Pharmaceutical Sciences, University of Douala, Douala, Cameroon; Faculty of Health Sciences, University of Bamenda, Bamenda, Cameroon

**Keywords:** Tobacco, Prevalence, College students, Africa

## Abstract

**Background:**

Tobacco is the most important avoidable risk for non communicable diseases. While tobacco consumption is stable or declining in developed countries, it is increasing in the developing world with a rate of 3.4 % per annum. The objective of this study was to estimate the prevalence and factors associated with cigarette smoking among college students.

**Methods:**

A cross-sectional study was conducted from December 2012 to April 2013 in secondary schools in Douala, Cameroon. A self-administered questionnaire was used to collect sociodemographic data, smoking behavior and peer smoking among college students. Logistic regression analyses was employed to identify factors associated with cigarette smoking.

**Results:**

Of a total of 2623 students included, 1579 (60.2 %) were female. The mean age of participants was 19.2 ± 2.53 years. The prevalence of current smoking was 11.2 % [95 % confidence interval (CI) 10 – 12], with 20 % in males and 5.3 % in females. Cigarette smoking was with significantly associated with friends smoking [Odds ratio (OR) 6.66; 95 % CI 4.69 – 9.45)], male gender (OR 3.61; 95 % CI 2.52 – 5.16), increase in age (OR 1.10; 1.03 – 1.17), parental smoking 1.69 (1.04 – 2.76), and attending general education (OR 1.85; 1.23 – 2.78).

**Conclusions:**

Cigarette smoking constitutes a significant health hazard in college students in Douala. Youth population and especially male students should be continuously targeted by preventive measures and sensitization campaigns against tobacco use. Parents should be aware on the influence of their smoking behavior on initiation of smoking in their children and should be encouraged to quit smoking.

## Background

Tobacco smoking remains a serious threat to global health, killing nearly 6 million people each year and causing excessive health-care costs and lost productivity [1]. About 80 % of the more than one billion smokers worldwide live in low- and middle-income countries, where the burden of tobacco-related illness and death is heaviest [1]. While tobacco consumption is stable or declining in developed countries, it is increasing in the developing world with a rate of 3.4 % per annum [2]. A recent study on projection of tobacco use predicted a worsening of tobacco epidemics in countries of Africa and eastern Mediterranean where health system are fragile [3]. Youth and women are the main targets of tobacco industries in these countries as they are developing economically [4]. Tobacco is the most important avoidable risk for non communicable diseases (NCDs) such as cancers, chronic lung disease, diabetes and cardiovascular diseases [1]. With the increasing prevalence of smoking in developing world over the years, NCDs will double the burden of infective and non-infective diseases [2].

The Global Youth Tobacco Survey (GYTS) which was designed by the Center for Disease Control and Prevention and the World Health Organization estimated the worldwide burden of tobacco use among youth [5]. The results of this survey which included school children from 131 countries showed a global prevalence of 8.9 % for current smoking students. This prevalence was highest in the WHO Region of the Americas (17.5 %) and the WHO European Region (17.9 %) and less than 10 % in the four other WHO regions [5]. In Ethiopia, the GYTS collaborative group reported 4.5 % of prevalence of smoking in males and 1 % in females aged 13–15 years [6]. In Cameroon, a country without any tobacco control legislation, the prevalence of smoking is relatively low [7]. The GYTS reported a cigarette smoking prevalence of 5.7 % among college students aged 13–15 years [8]. It has been shown that children who start smoking during a younger age are more likely to smoke as adults than individuals who begin at older ages [9]. Therefore, though it is important to determine the burden of tobacco use in this age group, it is also necessary to investigate its associated factors in order to design efficient preventive programs against tobacco consumption. Only few studies have investigated the factors associated with tobacco use among school adolescents in sub-Saharan Africa [10–12] and we didn’t find any related study in Cameroon. We therefore aimed in this study to estimate the prevalence and associated factors of cigarette smoking in college students.

## Methods

### Study design and setting

A cross-sectional study was conducted in secondary schools in Douala from 1^st^ December 2012 to April 30, 2013. Douala is the economic capital and the largest biggest city of Cameroon. It is also the headquarters of the Littoral region and the population was estimated at 1907479 inhabitants in 2005 [13]. In Cameroon, secondary education which follows the 6 years of primary education has a 7 years duration and is divided into 2 cycles: the 1^st^ cycle which comprises the 4 first years of studies and the 3 last years of the secondary education constitute the 2^nd^ cycle [14]. Students are allowed to choose technical and vocational education or general education. They can register either in a private or public school.

### Participants and sampling

We conveniently choose to include students attending the second cycle of secondary education. Of a total of 300 secondary schools in the city of Douala, 85 of them were excluded because they had only the first 4 years of secondary education. We finally had 215 eligible schools. Eligible students were those present in class during our visit in schools. Non consenting students were excluded.

We used a multistage probability proportional to size stratified sampling procedure with the school being the primary sampling unit. In the first stage, the strata were defined according to the type of education (general vs technical and vocational) and the status (public vs private). We thus had 4 strata: public general (20 schools), private general (118 schools), public technical and vocational (6 schools) and private technical and vocational (71 schools). These schools were randomly selected in proportion to their size, giving respectively 3, 20, 1 and 12 schools for the above strata. In the second stage, 2 classes were randomly selected in each school and finally, in each class, all students were invited to participate in the study.

The study was approved by the Cameroon’s National Ethics Committee. In addition, we got administrative authorizations from the Ministry of Secondary Education and the school authorities. Parents of students aged less than 18 years were also informed about the scope of the study and gave a verbal consent before the recruitment of their children in the study.

### Data collection

The selected students were given a self-administered anonymous questionnaire in their classroom during breaks. Teachers were asked to leave the classroom during the survey administration and the data collection was supervised by a trained investigator who was present in the class. He collected the forms as soon as they were filled by the students.

The questionnaire assessed the sociodemographic data (age, gender, year of education), number of classes repeated, smoking behavior (smoking status, reason of smoking, smoking during the last 30 days), smoking status of family members and friends, knowledge of tobacco hazards. For the latter, participants were asked to give a yes or no response for the question if they knew some negative effect of tobacco health, without giving the details of this hazards. The Fagerström score of nicotine dependence was accessed in smokers. A score smaller than 3 indicates low dependence while a score between 3 and 6 indicates moderate dependence and a score between 7 and 10 reflects a high nicotine dependence [15].

The dependent variable in this study was the smoking status. Were considered as current smokers in this study, students smoking at least one cigarette per month. Daily smokers were those smoking at least one cigarette per day, weekly smokers were those smoking 1 to 6 cigarettes per week, ,while occasional smokers or monthly smokers were those smoking 1 to 3 cigarettes per month. The exposure variables were: age, gender, type of education (general vs technical), school status (private vs public), number of classes repeated, parental smoking, peer smoking and knowledge of health effect of smoking.

### Statistical analysis

Data were entered and analyzed using IBM SPSS statistics Version 20 (Armonk, NY: IBM Corp). Descriptive statistics included frequencies and proportions for categorical data and means with standard deviations (SD) for continuous data. Logistic regression analysis was used to assess the factors associated with smoking. A univariate analysis was firstly performed to estimate crude odds ratios and their 95 % confidence intervals (95 % CI). Variables found to be significantly associated in univariate analysis were considered in the multivariable models using a stepwise backward elimination procedure. A *p*-value less than 0.05 was considered to be statistically significant.

## Results

### General characteristics of participants

Of a total of 3170 students who completed the survey, exploitable data were available for 2623 (82.7 %) respondents. Of them, 1579 (60.2 %) were female and 1044 (39.2 %) were male. The mean age of the participants was 19.2 ± 2.53 years (range 12 – 29). Students aged between 15 and 19 years were the most represented (55.7 %). The mean number of classes repeated by students was 1.28 ± 0.97. The other baseline characteristics of participants are showed in Table 1.

**Table 1:** General characteristics of participants (*n* = 2623)

Variables	Number (%)
Gender	
Male	1044 (39.8 %)
Female	1579 (60.2 %)
Age	
<15 years	45 (1.7 %)
15 - 19 years	1461 (55.7 %)
20 - 24 years	1057 (40.3 %)
25 - 29 years	60 (2.3 %)
Year of education	
5^th^	415 (15.8 %)
6^th^	1336 (50.9 %)
7^th^	872 (33.3 %)
Have ever smoked a cigarette	
Yes	879 (33.5 %)
No	1744 (66.5 %)
Have repeated a class	
Yes	1972 (75.2 %)
No	651 (24.8 %)
Type of education	
General	1868 (71.2 %)
Technical and vocational	755 (28.8 %)
School status	
Public	395 (15.1 %)
Private	2228 (84.9 %)
Knowledge of warmful effects smoking	
Yes	2070 (78.9 %)
No	553 (21.1 %)
Smoked the last 30 days	
Yes	270 (10.3 %)
No	2353 (89.7%)
Smoking status	
Non smoker	2330 (88.8 %)
Regular smoker	221 (8.4 %)
Occasional smoker	72 (2.8 %)

### Smoking status of participants

One third of the respondents (33.5 %) had ever smoked a cigarette while the prevalence of current cigarette smoking was 11.2 % (95 % CI 10 – 12) with 8.4 % being regular smokers and 2.8 % occasional smokers. The male prevalence (20 %) of cigarette smoking was significantly higher than that of females (5.3 %) (*P* = 0.000). The mean age at starting smoking of cigarettes was 14.6 ± 3.8 (range 6 – 24) and 270 (10.3 %) smoked at least one cigarette in the last 30 days prior the study. Among the 293 cigarette smokers, the Fagerström score for nicotine dependence was high for 18.4 % of them, moderate for 46.4 % and low for 35.2 %. The main reasons of starting smoking were curiosity (92.1 %), pleasure (91.8 %) and stressful situations (90.4 %). Table 2 shows the other attitudes of smoking students.

**Table 2:** Attitudes towards smoking of cigarette smoking students (*n* = 293)

Characteristics	Number (%)
Fagerström score	
Low (0–2)	103 (35.2 %)
Moderate (3–6)	136 (46.4 %)
High (7–10)	54 (18.4 %)
Place of smoking	
At home	78 (26.6 %)
In school	46 (15.6 %)
When with smokers	112 (38.2 %)
During recreative parties	231 (78.8 %)
Reason of starting smoking	
Imitation	254 (86.6 %)
Stress	265 (90.4 %)
Curiosity	270 (92.1 %)
Pleasure	270 (92.1 %)
Advertisement	151 (51.5 %)
Want to quit smoking	171 (58.3 %)
Reason of the desire to quit smoking (*n* = 171)	
Save money	121 (70.7 %)
Keep a good health	161 (94.1 %)
Self discipline	156 (91.2 %)
Have stopped smoking in the past	132 (45 %)

### Risk factors of cigarette smoking

The univariate analysis revealed that male sex, age, attending general education, number of classes repeated, parental smoking, family smoking and friends smoking were associated with current smoking status (Table 3). After adjusting these variables to each other in the multivariate analysis, the most important risk factors for cigarette smoking was friends smoking (OR 6.66; 95 % CI 4.69 – 9.45). It was followed by male sex (OR 3.61; 95 % CI 2.52 – 5.16). An increase in age was also associated with smoking as well as parental smoking and attending a general education system (Table 4).

**Table 3:** Univariate analysis of factors associated with current cigarette smoking among college students in Douala

Variables	Cigarette smoking		*P* value	Crude OR (95 % CI)
	Yes (*n* = 293)	No (*n* = 2330)		
Gender				
Male	209 (20 %)	836 (80 %)	0.000	4.45 (3.38 – 5.85)
Female (ref)	84 (5.3 %)	1494 (94.7 %)		
Age, per year increase			0.000	1.12 (1.07 – 1.18)
Type of education				
General school	239 (12.8 %)	1629 (87.2 %)	0.000	1.90 (1.39 – 2.59)
Technical school (ref)	54 (7.2 %)	701 (92.8 %)		
School status				
Public school	42 (10.6 %)	353 (89.4 %)	0.71	0.93 (0.66 – 1.32)
Private school (ref)	251 (11.3 %)	1977 (88.7 %)		
Number of class repeated			0.000	1.30 (1.13 – 1.48)
Parental smoking				
Yes	85 (25.4 %)	250 (74.6 %)	0.000	3.4 (2.55 – 4.51)
No (ref)	208 (9.1 %)	2080 (90.9 %)		
Family smoking				
Yes	133 (18.8 %)	576 (81.2 %)	0.000	2.84 (2.2 – 3.66)
No (ref)	160 (8.3 %)	1754 (91.6 %)		
Friends smoking				
Yes	242 (31.6 %)	523 (68.4 %)	0.000	9.9 (7.46 – 13.15)
No (ref)	51 (2.7 %)	1807 (97.3 %)		
Knowledge of warmful effects smoking				
Yes	233 (11.3 %)	1837 (88.7 %)	0.55	1.10 (0.79 – 1.51)
No (ref)	60 (10.9 %)	493 (89.1 %)

**Table 4:** Multivariate analysis of factors associated with cigarette smoking among college students in Douala

Variables	Cigarette smoking (*n* = 293)	aOR (95 % CI)	*P*-value
Gender			
Male	209 (20 %)	3.61 (2.52 – 5.16)	0.000
Female	84 (5.3 %)		
Age, per year increase	-	1.10 (1.03 – 1.17)	0.002
Type of education			
General school	239 (12.8 %)	1.85 (1.23 – 2.78)	0.003
Technical school	54 (7.2 %)		
Number class repeated, increase	-	1.04 (0.0.87 – 1.25)	0.62
Parental smoking			
Yes	85 (25.4 %)	1.69 (1.04 – 2.76)	0.03
No	208 (9.1 %)		
Family smoking			
Yes	133 (18.8 %)	1.48 (0.97 – 2.26)	0.06
No	160 (8.3 %)		
Friends smoking			
Yes	242 (31.6 %)	6.66 (4.69 – 9.45)	0.000
No	51 (2.7 %)		

## Discussion

In this survey on smoking habits of college students in Cameroon, we found that the prevalence of cigarette smoking among college students was 11.2 %. The main predictors of cigarette smoking were having friends who smoke, male sex, age, parental smoking and attending general education.

Whatever the case this prevalence is higher than 5.7 % found in GYTS survey in Cameroon in 2008 [8]. May be the prevalence of smoking is increasing with the years, but we should notice that the GYTS study involved adolescents of 13 to 15 years while our study included college students with an elder age. It has been demonstrated that the prevalence of smoking increases with age among youths [16–18]. Our prevalence is similar to that of a study conducted in Ethiopia among college students aged 15 to 25 years which showed 12.2 % of smokers [12].

Having smoking friends in this study was the most important independent factor associated with cigarette smoking. Reports from different regions of the world found similar results [17, 19–22]. Evidence from two longitudinal studies conducted in the United States showed that non-smoking adolescents who have friends who smoke are more likely to start smoking in the future than those without any smoking friends [23, 24]. We also found that parental smoking was associated with smoking among college students. The critical influence of parental smoking on adolescent’s smoking behavior was demonstrated by Bricker et al. in a cohort study involving five thousand families [25]. This finding is consistent with the results of other studies carried out in developing countries as well as in industrialized countries [26]. In fact, children are more likely to reproduce the behaviors and attitudes of their parents who are considered by them as models. Secondly, as demonstrated by Scragg et al., parents who smoke are more likely to allow smoking in the house [27]. Students living with another family member who smoke had a twofold risk of being smokers than those living with non-smokers. Similar results were recently found by Shadid and Hossain in Jordan [28].

In this study, the influence of smoking of peers on the smoking status of students was greater than that of parental smoking. Similar results were reported in previous studies in sub-Saharan Africa [6, 29]. On contrary, although a study conducted in 27 European countries reported parental smoking and smoking of peers as factors associated with smoking initiation, the effects of these two factors were similar [30].

Male sex was strongly associated with cigarette smoking. The same results have been found in other African countries [12, 22, 26] and in studies carried out in middle East and in South East Asia [26, 28, 31]. On the contrary, in developed countries, the disparity between male and female prevalence is smaller [32]. These data show the difference in social-cultural habits of different parts of the world. However, data concerning smoking prevalence in women in Africa may be underreported, especially in areas where smoking of women is not culturally accepted and is socially regarded as a pejorative behavior [26].

We detected an association between age and cigarette smoking in the present study. An increase in age was increasing the odds of smoking. Although this association has not been revealed by most of the studies, it was reported by some studies [12, 16, 18, 33]. One possible explanation for this finding is that during adolescence, the self-affirmation of adolescents and their risk behavior increase with increasing age. Consequently, at the late adolescence, there is a high risk of smoking [34, 35].

Attending general secondary education was another factor associated with cigarette smoking. The type of education as factor associated with smoking among students has not been well studied. However, our result is similar to that of Mohammad in Iran [36] but contrary to that of Nowicka-Sauer et al. in Poland who identified technical high school as a predictor of tobacco smoking [37]. Further studies are needed to investigate the role of technical or general education in the initiation of smoking among students.

The strengths of this study are the large sample of students and the sampling methods which participate to the accuracy of our results. This is confirmed by the narrowed confidence intervals that we obtained in the multivariate regression analysis.

Meanwhile, there are some limitations in this study. First, the prevalence of smoking may have been underestimated by negative responses from students who smoke secretly. Meanwhile, 17.3 % of students were excluded in this study because of they didn’t answer to key questions. The prevalence of smoking might have been increased or decreased if they had responded properly to these questions. In addition, some factors such as psychosocial factors, the influence of media were not assessed.

In conclusion, although the prevalence of smoking among college students in Douala is low, it may increase if there is no efficient action against tobacco use in Cameroon. Male sex, parental and peer smoking are the main predictors of smoking among youths. There is a need to design and implement effective preventive measures against tobacco use. In addition to college students, smoking families should be targeted by these programs.
